# Endothelial Dec1-PPARγ Axis Impairs Proliferation and Apoptosis Homeostasis Under Hypoxia in Pulmonary Arterial Hypertension

**DOI:** 10.3389/fcell.2021.757168

**Published:** 2021-10-26

**Authors:** Xiaoming Li, Chengcheng Liu, Wenwen Qi, Qiu Meng, Hui Zhao, Zhenxiao Teng, Runtong Xu, Xinhao Wu, Fangyuan Zhu, Yiming Qin, Miaoqing Zhao, Fenglei Xu, Ming Xia

**Affiliations:** ^1^Department of Otolaryngology, Shandong Provincial Hospital Affiliated to Shandong First Medical University, Jinan, China; ^2^Medical Science and Technology Innovation Center, Shandong First Medical University and Shandong Academy of Medical Sciences, Jinan, China; ^3^Department of Central Laboratory, Shandong Provincial Hospital Affiliated to Shandong University, Jinan, China; ^4^Department of Otolaryngology, Shandong Provincial Hospital, Cheeloo College of Medicine, Shandong University, Jinan, China; ^5^College of Chemical Engineering and Materials Science, Shandong Normal University, Jinan, China; ^6^Department of Pathology, Shandong Provincial Hospital Affiliated to Shandong First Medical University, Jinan, China

**Keywords:** hypoxia, differentiated embryo-chondrocyte expressed gene 1 (Dec1), peroxisome proliferative activated receptor-γ (PPARγ), proliferation, apoptosis, pulmonary arterial hypertension

## Abstract

**Background:** The hypoxia-induced pro-proliferative and anti-apoptotic characteristics of pulmonary arterial endothelial cells (PAECs) play critical roles in pulmonary vascular remodeling and contribute to hypoxic pulmonary arterial hypertension (PAH) pathogenesis. However, the mechanism underlying this hypoxic disease has not been fully elucidated.

**Methods:** Bioinformatics was adopted to screen out the key hypoxia-related genes in PAH. Gain- and loss-function assays were then performed to test the identified hypoxic pathways *in vitro*. Human PAECs were cultured under hypoxic (3% O_2_) or normoxic (21% O_2_) conditions. Hypoxia-induced changes in apoptosis and proliferation were determined by flow cytometry and Ki-67 immunofluorescence staining, respectively. Survival of the hypoxic cells was estimated by cell counting kit-8 assay. Expression alterations of the target hypoxia-related genes, cell cycle regulators, and apoptosis factors were investigated by Western blot.

**Results:** According to the Gene Expression Omnibus dataset (GSE84538), differentiated embryo chondrocyte expressed gene 1-peroxisome proliferative-activated receptor-γ (Dec1-PPARγ) axis was defined as a key hypoxia-related signaling in PAH. A negative correlation was observed between Dec1 and PPARγ expression in patients with hypoxic PAH. *In vitro* observations revealed an increased proliferation and a decreased apoptosis in PAECs under hypoxia. Furthermore, hypoxic PAECs exhibited remarkable upregulation of Dec1 and downregulation of PPARγ. Dec1 was confirmed to be crucial for the imbalance of proliferation and apoptosis in hypoxic PAECs. Furthermore, the pro-surviving effect of hypoxic Dec1 was mediated through PPARγ inhibition.

**Conclusion:** For the first time, Dec1-PPARγ axis was identified as a key determinant hypoxia-modifying signaling that is necessary for the imbalance between proliferation and apoptosis of PAECs. These novel endothelial signal transduction events may offer new diagnostic and therapeutic options for patients with hypoxic PAH.

## Introduction

As a complex, progressive, and lethal disease, pulmonary arterial hypertension (PAH) is characterized by the increased muscularity of pulmonary arteries (PAs), resistance to blood flow, irreversible right ventricular (RV) failure, and premature death (Humbert et al., 2018). Although its precise etiology is poorly understood, hypoxia has been identified as an important contributor to PAH (Stenmark et al., 2006). According to the Sixth World Symposium on Pulmonary Hypertension, PAH can be divided into five clinical groups. Hypoxic pulmonary hypertension (HPH) belongs to the third group and highly prevails in a wide range of hypoxia-associated diseases, such as chronic obstructive pulmonary diseases, obstructive sleep apnea (OSA), and high-altitude illness (Simonneau et al., 2014). The morbidity and mortality of PAH increase dramatically with OSA severity (Adir et al., 2021). This interaction can be explained by the hypoxia-induced endothelial dysfunction, which is critical in vascular remodeling and vasoconstriction (Sehgal and Mukhopadhyay, 2007; Houten, 2015).

Intermittent hypoxia, the physiological hallmark of OSA, increases oxidative stress in endothelial cells, which in turn results in endothelial dysfunction and remodeling (Bauters et al., 2016; Benjafield et al., 2019). The transcriptional factor peroxisome proliferative-activated receptor-γ (PPARγ) links these pathologies in vascular disease conditions and functions as a crucial mediator of the hypoxic response (Montaigne et al., 2021). As a ligand-activated nuclear hormone receptor, PPARγ can be stimulated in response to structurally diverse ligands and execute anti-inflammatory and antioxidant effects in many tissues to modulate cell differentiation, growth, inflammation, apoptosis, and angiogenesis (Polikandriotis et al., 2005). Insufficient PPARγ signaling is essential for PAH pathogenesis (Hansmann et al., 2008; Guignabert et al., 2009; Nisbet et al., 2010; Alastalo et al., 2011; Calvier et al., 2019), although the mechanisms have not been fully understood. PPARγ protein is highly expressed in the pulmonary vascular endothelium of normal individuals but is substantially decreased in the lungs and distal PAs of patients with PAH (Guignabert et al., 2009). PPARγ expression is also reduced in PASMCs and PAECs isolated from the PAs of HPH mice (Alastalo et al., 2011; Calvier et al., 2019). Mice with inducible deletion of PPARγ either in PAECs or PASMCs develop PAH (Hansmann et al., 2008). Pharmacological restoration of PPARγ with either pioglitazone or troglitazone shows therapeutic effects in preclinical animal models (Nisbet et al., 2010). The mechanism on how PPARγ is repressed under hypoxia in HPH must be explored.

In this study, the differentially expressed genes (DEGs) between PAH and normal control tissues were examined from the Gene Expression Omnibus (GEO) dataset (GSE84538), and the hypoxia-related DEGs for PAH were estimated. The differentiated embryo–chondrocyte-expressed gene 1 (Dec1), a hypoxia-induced transcriptional factor, was significantly upregulated in PAH. The protein–protein interaction (PPI) network was further established through the shared DEGs, and a Dec1-PPARγ interaction was found in PAH. Given that Dec1 is a hypoxia-regulated transcriptional factor and is involved in cell survival vs. death, the Dec1-PPARγ axis might be responsible for the proliferation promotion and apoptosis resistance of PAECs in hypoxic PAH.

## Materials and Methods

### Ethics

All experiments were approved by the Ethics Committee of Shandong Provincial Hospital Affiliated to Shandong First Medical University. Written informed consent was obtained from all the participants.

### Bioinformatic Analysis of Hypoxia-Related Differentially Expressed Genes in Patients With Pulmonary Arterial Hypertension

RNA-seq expression data of patients with PAH and the corresponding control subjects were extracted from the GEO database (GSE84538). These data were accessed by high throughput sequencing RNA from pulmonary arteries. The raw expression profiles were normalized in Deseq2 in the Affy installation package of R software, and the DEGs were screened out by the following selection criteria: *p* < 0.05 and |log_2_ fold change (FC)| > 1 (Rawal et al., 2021). Volcano plot and heatmaps were used to visualize the DEGs by using the R packages of ggplot2 (v3.3.0) and pheatmap, respectively (Wickham, 2009). The PPI networks were generated in Cytoscape (3.7.2) by the Search Tool for the Retrieval of Interacting Genes/Proteins database (StringDB^1^) protein query (Szklarczyk et al., 2019). Red and blue nodes represented the up and downregulated genes, respectively. GO functional enrichment analysis was performed by the R package “clusterProfiler,” and the top 20 of the most significant entries were obtained with a threshold of a *p*-value set at 0.05 (Yu et al., 2012).

### Enzyme-Linked Immunosorbent Assay

Peripheral venous blood was collected from patients suffering from OSA with or without PAH, then centrifuged for 10 min at 3,000 rpm, and stored in frozen aliquots at −80°C. Human-specific commercial ELISA assays were applied to evaluate the serum concentrations of Dec1 (Shuangying Biological Technology Co., Ltd., Shanghai, China) and PPARγ (X-Y Biotechnology, Shanghai, China) in accordance with the instructions of the manufacturer. Absorbance was measured at 450 nm using a microtiter plate reader. Experiments were repeated three times.

### Cell Culture

Human normal PAECs (hnPAECs) were purchased from Lonza (Basel, Switzerland) and cultured for three to six passages prior to use in the medium (EBM-2) provided by the manufacturers. PAH-PAECs were harvested from the explanted lung tissues of patients with PAH, and healthy-PAECs were obtained from the unused lungs of healthy donors. The isolated healthy- and PAH-PAECs were purified using CD31 antibody by the magnetic cell-sorting system (Miltenyi Biotec, Bergisch Gladbach, Germany) in accordance with the instructions of the manufacturer (Comhair et al., 2012). Cultured PAECs were placed in a standard normoxia incubator (21% O_2_, 5% CO_2_, 37°C) or a hypoxic incubator (3% O_2_, 5% CO_2_, 37°C). The hnPAECs were preconditioned with normoxia (pNor-PAECs) or hypoxia (pHyp-PAECs) for 1 week to observe the effect of hypoxia on the phenotype conversions of PAECs. In a separate experiment, the hnPAECs were treated with concentrations of various oxidative or inflammatory PAH stimuli (serum deprivation, H_2_O_2_ for 100 μM, TGF-β for 20 ng/ml, and IL-6 for 50 ng/ml) for 6, 12, and 24 h *in vitro*.

### Construction and Transduction of Recombinant Lentiviral Vectors

Plasmids with Dec1 overexpression (Ov-Dec1), PPARγ overexpression (Ov- PPARγ), and empty control vectors (Ov-Ctrl) were generated as previously described (Li et al., 2016). Plasmids with the short-hairpin RNA of Dec1 (Sh-Dec1, sequence: 5′-GGACTCTTCCTTAATTGCGCC-3′) and nontargeting plasmids (Sh-Ctrl) were generated and recombined into the psi-LVRU6MP vectors in accordance with the instructions of the manufacturer (GeneCopoeia, Rockville, MD, United States). The plasmids were transfected by Lipofectamine 2000 following the instructions of the manufacturer (Thermo Fisher Scientific, San Jose, CA, United States).

### Cell Counting Kit 8 Assessment and Flow Cytometry

Cell survival was measured by the CCK8 in accordance with the recommended instructions of the manufacturer (APExBIO, Houston, TX, United States), and cell apoptosis was detected by flow cytometry. After transfection or treatment with hypoxia for the indicated times, the PAECs were collected and double-stained with Annexin V-FITC and propidium iodide by using Annexin V-FITC Apoptosis Detection Kit I (Biosciences Pharmingen, CA, United States) following the instructions of the manufacturer. Apoptosis was measured and analyzed by a FACScan system (Becton Dickinson, Heidelberg, Germany). The cells with Annexin V-FITC positive and PI negative were considered as early apoptosis, and those with Annexin V-FITC positive and PI positive were considered as late apoptosis or necrosis. These experiments were repeated three times.

### Ki-67 Immunofluorescence Staining

Ki-67 immunofluorescence staining was performed using the labeled streptavidin–biotin method to evaluate the proliferation of PAECs. After being washed three times with phosphate buffer saline (PBS), the PAECs were sequentially fixed in 4% paraformaldehyde at 4°C for 20 min, permeabilized with 0.2% Triton X-100 in PBS for 5 min, and blocked with 3% bovine serum albumin in PBS for 1 h at room temperature. The cells were then incubated with the Ki-67 primary antibodies (1:500 dilution, Abcam, Cambridge, MA, United States) at 4°C overnight, and then stained with goat anti-rabbit IgG antibody (1:500, Thermo Fisher Scientific, Alexa Fluor 488) at 37°C for 1 h. Finally, nuclei were stained with 4′,6-diamidino-2-phenylindole (DAPI; 1:500, Abcam). The Ki-67 immunostaining of PAECs were detected by a fluorescence microscope (Zeiss, Heidenheim, Germany). The percentage of Ki-67-positive cells was indicated as the proliferation rate of PAECs. Measurements were repeated three times.

### Quantitative Real-Time Polymerase Chain Reaction

Total RNA of PAECs was extracted in accordance with the instructions of the Trizol reagent (Invitrogen), and reverse transcription was performed using reverse transcriptase (Thermo Fisher Scientific, Inc.) as reported previously (Zhao et al., 2017). The primers were designed and obtained from Sangon Biotech Co. (Shanghai, China) as follows: Dec1, forward primer 5′-GAAAGGATCGGCGCAATTAA-3′, reverse primer 5′-CATCATCCGAAAGCTGCATC-3′; and β-actin, forward primer 5′-TGGCACCACACCTTCTACAA-3′, reverse primer 5′-GCAGCTCGTAGCTCTTCTCC-3′. The relative level of mRNA was calculated by the 2^–Δ^^Δ^^CT^ method (Teng et al., 2012). Fold change of the relative mRNA expression was evaluated as the average fold change of the hypoxic PAECs compared with that of the normoxic PAECs. Measurements were repeated three times.

### Western Blot

Proteins were extracted from human pulmonary artery tissues or PAECs and lysed in RIPA lysis buffer (RIPA, Beyotime, China) in accordance with the instructions of the manufacturer. Protein concentrations were evaluated using the BCA assay based on the albumin standard. The primary antibodies were used as follows: Dec1 and PPARγ (Abcam, United States, dilution: 1/1,000, for both), Cyclin B1 and Cyclin D1 (Sigma, United States, dilution 1/1,000, for both), Bax (Cell Signaling Technology, United States, dilution: 1/1,000), Bcl-2 (Sigma, United States, dilution 1/1,000), cleaved caspase 3, and β-actin (Santa Cruz, United States, dilution 1/1,000, for both). Detailed information of Western blot was described previously (Li et al., 2020). β-actin was used as a loading control. Fold change of protein expression was evaluated as average fold change for the ratio of targeting genes/β-actin in treated samples compared with that in the controls. Measurements were repeated three times.

### Statistical Analysis

All data were presented as mean ± SEM and independently repeated three times. SPSS 13.0 software (SPSS, Chicago, IL, United States) was applied for statistical analysis. GraphPad Prism 6 (GraphPad Software) was used to draw charts. Student’s *t*-test was used for data comparison with the two groups. One-way or two-way ANOVA was performed for multiple comparisons. A value of *p* < 0.05 was considered to be statistically significant.

## Results

### Identification and Validation of Differentiated Embryo-Chondrocyte-Expressed Gene 1-Peroxisome Proliferative-Activated Receptor-γ Axis by Bioinformatic Analysis in Patients With Pulmonary Arterial Hypertension

**FIGURE 1:**
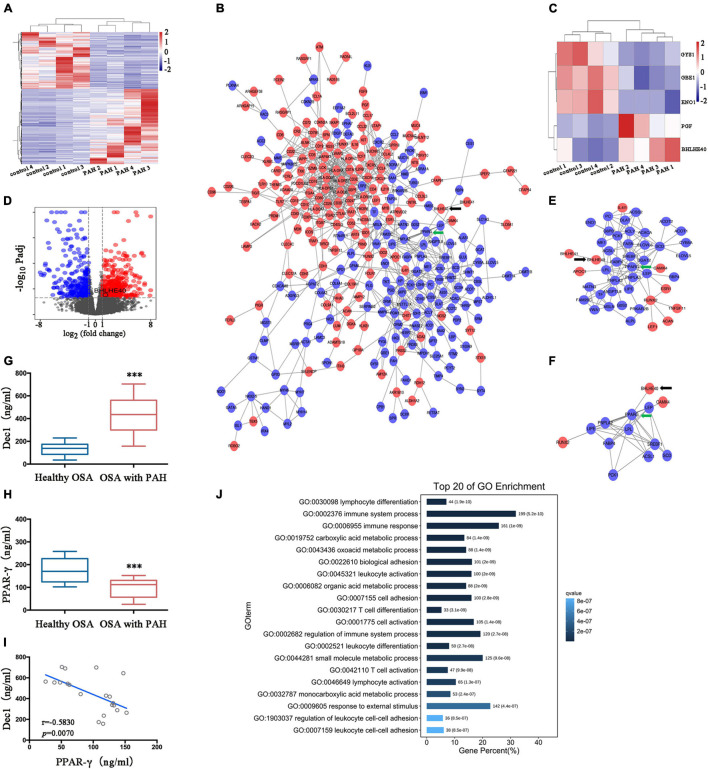
The differentiated embryo-chondrocyte expressed gene 1-peroxisome proliferative activated receptor-γ (Dec1-PPARγ) axis is dysregulated in patients with PAH. **(A)** The heatmap of differentially expressed genes (DEGs) in patients with pulmonary arterial hypertension (PAH) from the GSE84538 database. **(B)** The protein-protein interactions (PPI) network of the DEGs. Black and green arrows indicate BHLHE40 (also named Dec1) and PPARγ, respectively. **(C)** The heatmap of the differentially expressed hypoxia-related genes in patients with PAH from the GSE84538 database. **(D)** The volcano plot showed the DEGs (upregulated in red and downregulated in blue) in patients with PAH from the GSE84538 database. **(E,F)** The PPI networks based on BHLHE40 (arrowed as black) and PPARγ (arrowed as green), respectively. **(G,H)** Plasma protein levels of Dec1 (G) and PPARγ **(H)** were determined by Enzyme-linked immunosorbent assay (ELISA) in obstructive sleep apnea (OSA) patients with or without PAH. One-way ANOVA tests were applied for statistical analysis. **(I)** Spearman’s correlation analysis was performed to evaluate the correlations between Dec1 and PPARγ expressions in OSA patients combined with PAH. **(J)** The top 20 entries of the GO enrichment analysis of the gene set. ^∗∗∗^*p* < 0.001.

Gene expression data in eight lung tissue samples (four patients with PAH and four healthy control subjects) were extracted from the GEO (GSE84538) database. Deseq2 analysis identified 756 DEGs, including 446 upregulated (represented in red) and 310 downregulated genes (represented in blue) between the PAH and normal control samples (Figure 1A). With the use of STRING, the PPI network revealed 890 edges and 385 nodes in these DEGs (Figure 1B). These screened DEGs with all known hypoxia-related genes were overlapped to determine which DEGs have important functions in HPH. As shown in Figure 1C, five hypoxia-related genes showed differential expression in PAH. Among them, BHLHE40 (also named Dec1) and placental growth factor (PGF) were upregulated, whereas ENO1 (enolase 1), glycogen branching enzyme 1 (GBE1), and glycogen synthase 1 (GYS1) were downregulated. The volcano plot represented all the DEGs with statistical significance (upregulated and downregulated genes represented in red and blue, respectively) and indicated the prominently upregulated BHLHE40 in PAH (Figure 1D).

As the most robust hypoxia-related DEGs in PAH (Figures 1B,C), BHLHE40 was selected as a target gene for further study. The PPI network based on the DEGs showed a potential connection between BHLHE40 and PPARγ, a well-known mediator of PAH and a core DEG in the PPI network (as schematically highlighted by a black or green arrow, Figure 1B). This connection was also observed in the PPI network based on BHLHE40 and PPARγ (as schematically highlighted by a black or green arrow, Figures 1E,F). All these results indicated that the dysregulation of Dec1-PPARγ axis might play major roles in PAH development. However, the expression levels of Dec1 and PPARγ in HPH were still unclear. Therefore, the expression status of Dec1 and PPARγ in OSA patients with or without PAH (*n* = 20 for both groups) was further determined by ELISA. Compared with that in the healthy OSA group, Dec1 was significantly increased (445.8 ± 38.92 vs. 133.7 ± 12.71, *p* < 0.001, Figure 1G), and PPARγ was decreased (146.3 ± 13.22 vs. 261.3 ± 16.97, *p* < 0.001, Figure 1H) in OSA patients with PAH. A negative correlation was found between Dec1 and PPARγ (*r* = −0.5830, *p* = 0.0070, Figure 1I). The negative transcriptional regulating effect of Dec1 on PPARγ has been previously reported. The potential molecular biological role of the Dec1–PPARγ axis was further uncovered by GO enrichment analysis (Figure 1J). These data indicated a close relationship between the Dec1–PPARγ axis and the progression of OSA-associated PAH.

### Pulmonary Arterial Hypertension Disrupts the Differentiated Embryo–Chondrocyte-Expressed Gene 1-Peroxisome Proliferative-Activated Receptor-γ Axis in Pulmonary Arterial Endothelial Cells

**FIGURE 2:**
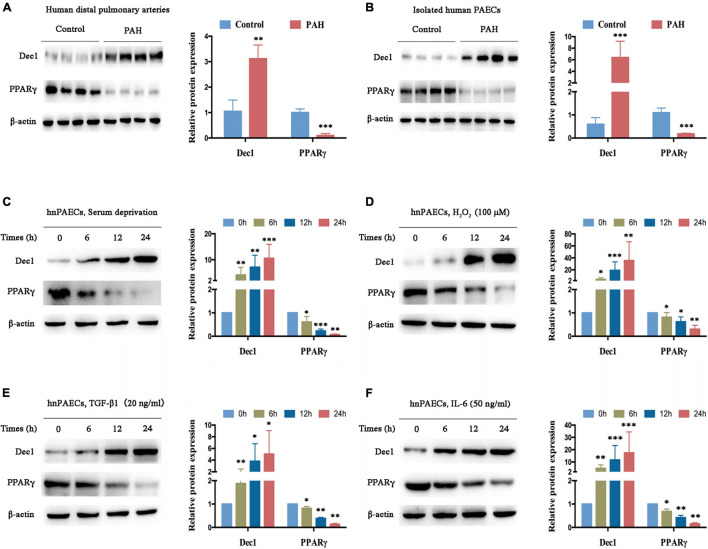
Dec1 is upregulated and PPARγ is downregulated in PAH. **(A)** Expression levels of Dec1 and PPARγ were determined by western blot in pulmonary arteries from PAH (*n* = 4) and healthy control patients (*n* = 4). **(B)** Human pulmonary arterial endothelial cells (PAECs) were isolated from PAH and healthy control patients (*n* = 4 per group), and western blot was performed to determine expressions of the Dec1–PPARγ axis. **(C–F)** Human normal PAECs (hnPAECs) were treated with serum deprivation **(C)**, 100 μM H_2_O_2_ **(D)**, 20 ng/ml TGF-β1 **(E)**, and 50 ng/ml IL-6 **(F)** *in vitro*. Expression levels of Dec1 and PPARγ in PAECs were determined by western blot (left part) at the indicated times. One-way ANOVA test was applied for statistical analysis (right part). Data are represented as mean ± SEM. Experiments were repeated for three independent times. ^∗^*p* < 0.05; ^∗∗^*p* < 0.01; ^∗∗∗^*p* < 0.001.

The expression of Dec1-PPARγ axis in human distal pulmonary arteries (PAs) was measured in individuals with or without PAH (control group, *n* = 4; PAH group, *n* = 4) for further understanding. Compared with the PAs in patients without PAH (used as control), Dec1 expression was significantly upregulated (>threefold increase, *p* < 0.01), and PPARγ expression was downregulated in the PAs of patients with PAH (Figure 2A, >fivefold decrease, *p* < 0.001). The protein levels of the Dec1–PPARγ axis were further examined in isolated PAECs from patients with or without PAH (*n* = 4 per group). A similar expression trend of the Dec1–PPARγ axis was found in the isolated PAECs. As shown in Figure 2B, the protein levels of Dec1 were significantly increased (>sixfold increase, <0.001), whereas those of PPARγ were decreased in PAH-PAECs compared with those in the control-PAECs (>fivefold decrease, *p* < 0.001). These results suggest that PAH exhibits disrupted expression of the Dec1-PPARγ axis.

The hnPAECs were cultured and treated with different PAH stimuli *in vitro* to further determine whether the disrupted expression of the Dec1-PPARγ axis is restricted to the presence of PAH. These stimuli promote PAH *via* oxidative stress and inflammation (Steiner et al., 2009; Wang et al., 2019; Reyes-Palomares et al., 2020). Consistent with the results for distal PAs and PAECs from patients with PAH, Dec1 was elevated, but PPARγ was declined in a time-dependent manner when the cells were treated with serum deprivation (Figure 2C), H_2_O_2_ (Figure 2D), TGF-β1 (Figure 2E), and IL-6 (Figure 2F). These findings indicate that the Dec1–PPARγ axis can also be disrupted by the PAH stimuli *in vitro* and may play critical roles in PAH progression.

### Differentiated Embryo–Chondrocyte-Expressed Gene 1 Modulates the Survival of Pulmonary Arterial Endothelial Cells Under Hypoxia

**FIGURE 3:**
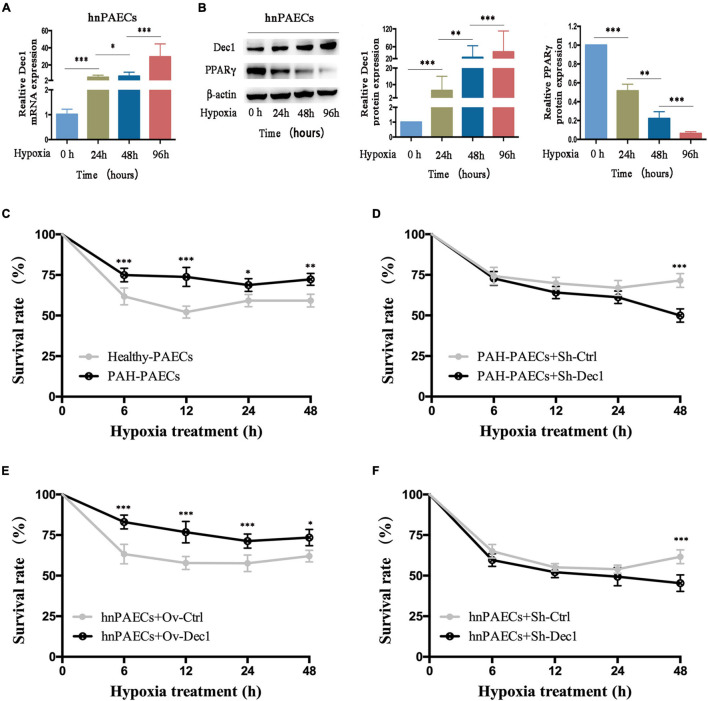
Effect of Dec1 on PAECs survival under hypoxia. **(A,B)** Human normal PAECs (hnPAECs) were treated with 3% O_2_ for 0, 24, 48, and 96 h and then performed real-time polymerase chain reaction (RT-PCR) or Western blot to determine the mRNA and protein levels of the target genes, respectively. **(A)** mRNA expression levels of Dec1. **(B)** Protein expression levels of Dec1 and PPARγ. One-way ANOVA test was applied for statistical analysis. **(C–F)** After 12 h of transient transfection, cells were treated with 3% O_2_ for 6, 12, 24, and 48 h and monitored the cell viability vs. time by the cell counting kit 8 (CCK-8) assay. Cells were plotted cell viability at the indicated times. **(C)** Healthy- and PAH–PAECs with no transfections. **(D)** PAH–PAECs transfected with Dec1-shRNA (Sh-Dec1) or the scrambled RNA (Sh-Ctrl) plasmids. **(E)** hnPAECs transfected with Dec1 overexpression (Ov-Dec1) or the empty control (Ov-Ctrl) plasmids. **(F)** hnPAECs transfected with Dec1–shRNA (Sh-Dec1) or the scrambled RNA (Sh-Ctrl) plasmids. Two-way ANOVA test was applied for statistical analysis. Data are represented as mean ± SEM. Experiments were repeated for three independent times. ^∗^*p* < 0.05; ^∗∗^*p* < 0.01; ^∗∗∗^*p* < 0.001.

Differentiated embryo-chondrocyte expressed gene 1 is dysregulated by hypoxia-driven responses and plays important roles in cell death and survival (Park and Park, 2012; Sato et al., 2016; Jia et al., 2018; Nakashima et al., 2018; Le et al., 2019). Therefore, the hnPAECs were treated with hypoxia. The results showed that the mRNA and protein levels of Dec1 were gradually increased in the hnPAECs with the extent of hypoxia (Figures 3A,B). However, the protein levels of PPARγ showed the opposite trend (Figure 3B). These results suggested that the Dec1–PPARγ axis was also reprogrammed by hypoxia in PAECs.

The question of how PAECs can overcome hypoxia to survive has been extensively investigated. In this work, whether the dysregulated Dec1–PPARγ axis contributes to the cell survival under hypoxia in PAECs was further explored. Cell survival was determined by the CCK-8 assay. As shown in Figure 3C, over 75% of the isolated PAH-PAECs survived comparing with the healthy-PAECs (60%) under hypoxia. These results showed that the PAH–PAECs have a significant pro-surviving ability in hypoxia environment. The transfected PAH–PAECs with Dec1–shRNA were studied to further illustrate whether Dec1 is essential for the survival of PAH–PAECs under hypoxia. The results showed that the survival rate of these cells (PAH–PAECs + Sh-Dec1) declined 25% compared with that of the controlled cells (PAH–PAECs + Sh-Dec1, Figure 3D). Therefore, Dec1 is responsible for the pro-surviving phenotype of PAH–PAECs under hypoxia. Dec1 was overexpressed or silenced in hnPAECs to further specify the role of Dec1 in this pro-surviving phenotype. The results showed the hnPAECs with Dec1 overexpression (hnPAECs + Ov-Dec1) under hypoxia showed an increase survival of up to 20% compared with the hnPAECs transfected with empty vectors (hnPAECs + Ov-Ctrl, Figure 3E). However, under half of the hnPAECs with deletion of Dec1 (hnPAECs + Sh-Dec1) survived compared with the controlled hnPAECs (65%, hnPAECs + Sh-Ctrl, Figure 3F) under hypoxia. All these data showed that Dec1 conferred to the ability of PAECs to survive under hypoxia.

### Hypoxia Recapitulates the Pro-proliferative and Anti-apoptotic Phenotypes of Pulmonary Arterial Endothelial Cells

**FIGURE 4:**
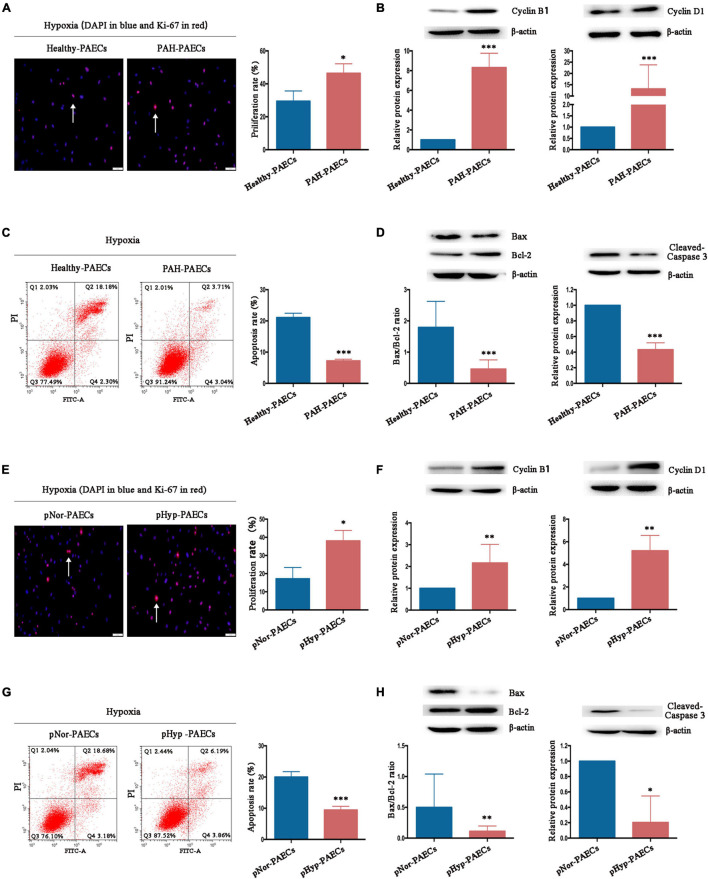
Effect of hypoxia on phenotypes of PAECs. Isolated healthy- or PAH–PAECs were treated with hypoxia (3% O_2_) for 24 h. Human normal PAECs (hnPAECs) were preconditioned with normoxia (21% O_2_, pNor-PAECs) or hypoxia (3% O_2_, pHyp-PAECs) for 1 week. Then these cells were assessed proliferation and apoptosis by immunofluorescence staining of Ki-67 and flow cytometry under hypoxia (3% O_2_), respectively. In proliferation assay, Ki-67 positive cells (red) with nuclear DAPI counterstain (blue) indicated the proliferating cells. In apoptosis map, cells were divided into four subpopulations: necrotic cells (Q1: annexin V FITC–/PI+), late apoptotic cells (Q2: annexin V FITC+/PI+), live cells (Q3: annexin V FITC–/PI–), and early apoptotic cells (Q4: annexin V FITC+/PI–). The proliferative (Cyclin B1 and Cyclin D1) and apoptotic factors (Bax, Bcl-2, and cleaved-caspase 3) were detected by Western blot. **(A)** Immunofluorescence staining of Ki-67 (left part, scale bars = 50 μm) and the proliferation rate (right part). **(B)** Expressions of Cyclin B1 and Cyclin D1. **(C)** Flow cytometry (left part) and the apoptosis rate (right part). **(D)** Expressions of Bax, Bcl-2, and cleaved caspase 3. **(E)** Immunofluorescence staining of Ki-67 (left part, scale bars = 50 μm) and the proliferation rate (right part). **(F)** Expressions of Cyclin B1 and Cyclin D1. **(G)** Flow cytometry (left part) and the apoptosis rate (right part). **(H)** Expressions of Bax, Bcl-2, and cleaved caspase 3. One-way ANOVA test was applied for statistical analysis. Data are represented as mean ± SEM. Experiments were repeated for three independent times. ^∗^*p* < 0.05; ^∗∗^*p* < 0.01; ^∗∗∗^*p* < 0.001.

The imbalance between proliferation and apoptosis, which is considered as the PAH phenotype of PAECs, is a major hallmark of the surviving PAECs in PAH development. Therefore, the effects and mechanisms of hypoxia on proliferation and apoptosis of PAECs were investigated. Isolated healthy- or PAH–PAECs were treated with hypoxia (3% O_2_) for 24 h, and their proliferation and apoptosis were determined by Ki-67 immunofluorescence staining and flow cytometry, respectively. Under hypoxia, the proliferation rate was higher in PAH-PAECs (46.45 ± 3.32) than in the healthy-PAECs (29.50 ± 3.57, *p* < 0.05, Figure 4A). Furthermore, the expression levels of proliferative factors, such as cyclin B1 and cyclin D1, were significantly upregulated in the PAH–PAECs compared with that in the healthy-PAECs (Figure 4B). By contrast, the apoptosis rate was lower in the PAH-PAECs than in the healthy-PAECs under hypoxia (7.22 ± 0.31 vs. 21.08 ± 0.80, *p* < 0.001, Figure 4C). The apoptotic indicators of Bax/Bcl-2 ratio and cleaved caspase-3 were significantly declined in the PAH-PAECs compared with that in the healthy-PAECs (Figure 4D). These data indicated a pro-proliferation and anti-apoptosis phenotypes of PAH-PAECs in hypoxic environments.

The hnPAECs were preconditioned under normoxia or hypoxia for 1 week to fully understand the influence of hypoxia in PAH progression. Compared with the normoxia-preconditioned PAECs (pNor-PAECs), the hypoxia-preconditioned PAECs (pHyp-PAECs) showed a significantly increased proliferation rate (38.05 ± 3.32 vs. 17.21 ± 3.57, *p* < 0.05, Figure 4E) but a decreased apoptosis rate (9.43 ± 0.68 vs. 20.01 ± 0.98, *p* < 0.001, Figure 4G) when cultured under hypoxia environment. In parallel with these data, the proliferative factors (Cyclin B1 and Cyclin D1) increased, but the apoptotic indicators (Bax/Bcl-2 ratio and cleaved caspase-3) decreased in the pHyp-PAECs compared with that of the pNor-PAECs (Figures 4F,H). Therefore, pHyp-PAECs had a PAH-like phenotype that exhibited a pro-proliferative and anti-apoptotic characteristic under hypoxia. These results were similar to those observed in PAH-PAECs and suggested that hypoxia environment promotes the pro-proliferative and anti-apoptotic phenotypes of PAECs.

### Differentiated Embryo–Chondrocyte-Expressed Gene 1 Is Essential for the Hypoxia-Induced Pulmonary Arterial Hypertension Phenotypes of Pulmonary Arterial Endothelial Cells

**FIGURE 5:**
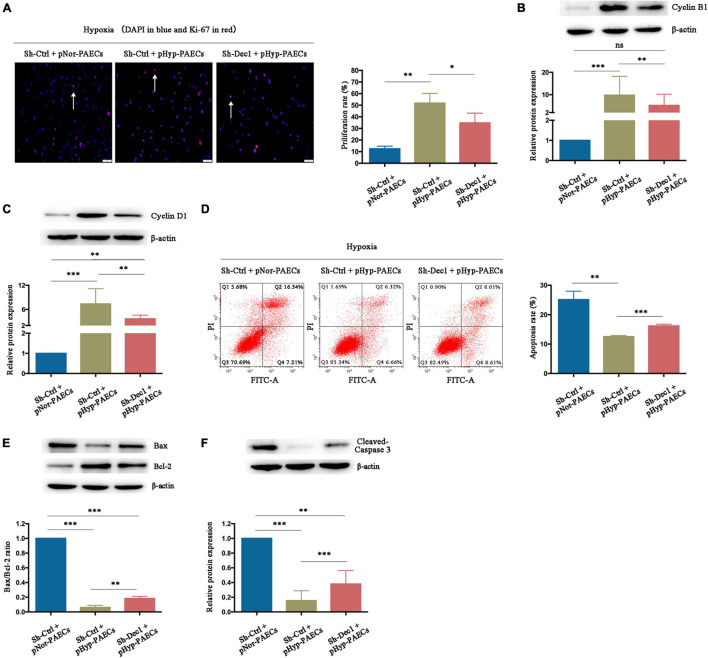
Hypoxia promotes the PAH phenotypes of PAECs by induction of Dec1. Human normal PAECs (hnPAECs) were transfected with Dec1–shRNA (Sh-Dec1) or scrambled RNA (Sh-Ctrl) and then preconditioned with normoxia (21% O_2_, pNor-PAECs) or hypoxia (3% O_2_, pHyp-PAECs) for 1 week. Next, these cells were assessed proliferation and apoptosis by immunofluorescence staining of Ki-67 and flow cytometry under hypoxia (3% O_2_), respectively. In proliferation assay, Ki-67-positive cells (red) with nuclear DAPI counterstain (blue) indicated the proliferating cells. In apoptosis map, cells were divided into four subpopulations: necrotic cells (Q1: annexin V FITC–/PI+), late apoptotic cells (Q2: annexin V FITC+/PI+), live cells (Q3: annexin V FITC–/PI–), and early apoptotic cells (Q4: annexin V FITC+/PI–). The proliferative (Cyclin B1 and Cyclin D1) and apoptotic factors (Bax, Bcl-2 and cleaved-caspase 3) were also detected by western blot. **(A)** Immunofluorescence staining of Ki-67 (left part, scale bars = 50 μm) and the proliferation rate (right part). **(B,C)** The expressions of Cyclin B1 **(B)** and Cyclin D1 **(C)**. **(D)** Flow cytometry (left part) and the apoptosis rate (right part). **(E,F)** The expressions of Bax, Bcl-2 **(E)**, and cleaved caspase 3 **(F)**. One-way ANOVA test was applied for statistical analysis. Data are represented as mean ± SEM. Experiments were repeated for three independent times. ^∗^*p* < 0.05; ^∗∗^*p* < 0.01; ^∗∗∗^*p* < 0.001.

Differentiated embryo-chondrocyte expressed gene 1 plays an important role in cell proliferation and apoptosis under various oxidative stress and inflammatory stimuli (Seino et al., 2015; Jia et al., 2018; Ming et al., 2018; Le et al., 2019). Therefore, the effects of Dec1 on proliferation and apoptosis of PAECs under hypoxia were examined. As shown in Figure 5A, the pHyp-PAECs were characterized by an increased proliferation rate compared with the pNor-PAECs (51.46 ± 1.89 vs. 12.44 ± 1.31, *p* < 0.01). The pHyp-PAECs with Dec1 knockdown (Sh-Dec1 + pHyp-PAECs) showed a lower proliferation rate than the corresponding control cells (Sh-Ctrl + pHyp-PAECs, 31.43 ± 3.36, *p* < 0.05) under hypoxia. Similarly, the expression levels of Cyclin B1 and Cyclin D1 were also upregulated by hypoxia but attenuated by Dec1 silencing (Figures 5B,C). In addition, a decrease in the apoptosis rate was also found in the pHyp-PAECs compared with that of the pNor-PAECs (12.54 ± 0.23 vs. 25.13 ± 1.65, *p* < 0.05). However, the apoptosis rate was reversed in pHyp-PAECs with Dec1 knockdown compared with that of corresponding control cells under hypoxia (12.54 ± 0.23 vs. 16.18 ± 0.34, *p* < 0.001, Figure 5D). In parallel, the decreased apoptotic indicators (Bax/Bcl-2 ratio and cleaved caspase-3) induced by the preconditioned hypoxia was also restored by Dec1 knockdown in PAECs (Figures 5E,F). Further comparison was conducted on the expression levels of the proliferative and apoptotic factors between Sh-Ctrl + pNor-PAECs and Sh-Dec1 + pHyp-PAECs groups. Cyclin D1, Bax/Bcl-2 ratio and cleaved caspase-3, but not Cyclin B1, showed significant expression (Figures 5B,C,E,F). These results indicated that knockout of Dec1 under hypoxia rescued the expression of Cyclin B1 normal to the greatest extent. Therefore, hypoxia induces the proliferation promotion and apoptosis resistance of PAECs, a PAH-like phenotype, which is regulated by Dec1.

### Differentiated Embryo–Chondrocyte-Expressed Gene 1 Promotes the Pulmonary Arterial Hypertension Phenotype of Human Pulmonary Arterial Endothelial Cells *via* Inhibition of Peroxisome Proliferative-Activated Receptor-γ

**FIGURE 6:**
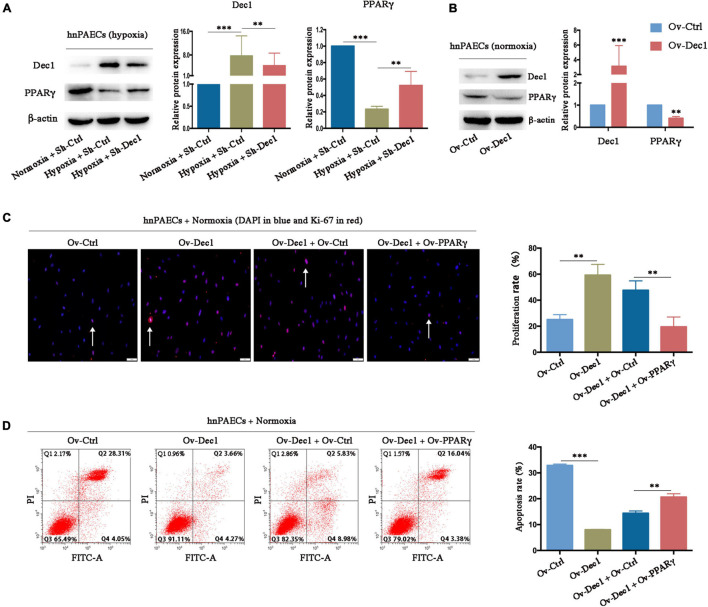
Dec1 promotes the PAH phenotypes of PAECs by inhibition of PPARγ. **(A)** Human normal PAECs (hnPAECs) were transfected with Dec1 Sh-RNA (Sh-Dec1) or the scrambled control (Sh-Ctrl) plasmids and then treated with normoxia (21% O_2_) or hypoxia (3% O_2_) for 24 h. Protein levels of Dec1 and PPARγ were detected by Western blot in the indicated cells. **(B)** hnPAECs were transfected with Dec1 overexpression (Ov-Dec1) or the empty control (Ov-Ctrl) plasmids and treated with normoxia (21% O_2_) for 24 h. Protein levels of Dec1 and PPARγ were detected by western blot in the indicated cells. **(C,D)** Human normal PAECs (hnPAECs) were transfected with Ov-Ctrl, Ov-Dec1, Ov-Dec1 + Ov-Ctrl, and Ov-Dec1 + Ov-PPARγ plasmids and treated with normoxia (21% O_2_) for 24 h. Then these cells were assessed proliferation and apoptosis by immunofluorescence staining of Ki-67 (**C**, scale bars = 50 μm) and flow cytometry **(D)**, respectively. In proliferation assay, Ki-67-positive cells (red) with nuclear DAPI counterstain (blue) indicated the proliferating cells. In apoptosis map, cells were divided into four subpopulations: necrotic cells (Q1: annexin V FITC–/PI+), late apoptotic cells (Q2: annexin V FITC+/PI+), live cells (Q3: annexin V FITC–/PI–), and early apoptotic cells (Q4: annexin V FITC+/PI–). One-way ANOVA test was applied for statistical analysis. Data are represented as mean ± SEM. Experiments were repeated for three independent times. ^∗^*p* < 0.05; ^∗∗^*p* < 0.01; ^∗∗∗^*p* < 0.001.

Peroxisome proliferative activated receptor-γ, which was downregulated in experimental PAH models and clinical patients with PAH, has been widely proven as a protective factor for PAH (Crossno et al., 2007; Hansmann et al., 2007, 2008; Guignabert et al., 2009; Alastalo et al., 2011; Gong et al., 2011; Calvier et al., 2017, 2019). PPARγ expression in hnPAECs was detected by gain- and loss-function assays to understand whether hypoxia-induced downregulation of PPARγ, at least partially, is associated with Dec1. As shown in Figure 6A, PPARγ was significantly downregulated by hypoxia but partially restored by Dec1 deletion. Under normoxia, the protein level of PPARγ was also decreased in Dec1-overexpressing hnPAECs compared with that in the corresponding control PAECs (Figure 6B), suggesting that PPARγ suppression was a direct result of Dec1 upregulation. These results confirmed that PPARγ expression is negatively regulated by Dec1 under hypoxia.

The hnPAECs were co-transfected with Dec1 and PPARγ overexpressing vectors and treated under normoxia to further clarify whether Dec1 upregulation alone is responsible for the PAH phenotype of PAECs. PAECs transfected with empty vectors was as control (Ov-Ctrl PAECs). Compared with that in Ov-Ctrl PAECs, Dec1 overexpression significantly increased the proliferation rate (59.26 ± 4.77 vs. 25.13 ± 2.17, *p* < 0.01, Figure 6C) and decreased the apoptosis rate of hnPAECs (8.040 ± 0.08 vs. 32.88 ± 0.30, *p* < 0.001, Figure 6D) under hypoxia. Furthermore, the proliferation rate of hnPAECs with Dec1 overexpression (Ov-Dec1 + Ov-Ctrl) was higher than that of the hnPAECs with co-overexpression of Dec1 and PPARγ (Ov-Dec1 Ov- PPARγ, 47.63 ± 4.16 vs. 19.51 ± 4.39, *p* < 0.01, Figure 6C). However, the apoptosis rate of hnPAECs with Dec1 overexpression (Ov-Dec1 + Ov-Ctrl) was significantly lower than that of the hnPAECs with co-overexpression of Dec1 and PPARγ (14.40 ± 0.51 vs. 20.67 ± 0.72, *p* < 0.01, Figure 6D). These results indicated that the increased proliferation rate and the decreased apoptosis rate induced by Dec1 overexpression were at least partially attributed to PPARγ downregulation.

## Discussion

Hypoxia and hypoxia-related pathways contribute to the vascular remodeling of PAH, which is characterized by the phenotype conversions of endothelial proliferation and apoptosis. Under hypoxic environment, the hallmark of OSA, reprogram occurs for hypoxia-responsive genes causing the survival of hyper-proliferative and anti-apoptotic PAECs and the structural changes of PAs. Although the dysfunctions of PPARγ signaling are closely correlated with PAH progression (Hansmann et al., 2008; Alastalo et al., 2011; Gong et al., 2011; Calvier et al., 2017, 2019), the underlying mechanisms by which hypoxia-responsive genes dysregulate PPARγ to trigger the endothelial phenotypic conversions have remained elusive. In this study, bioinformatic analysis revealed that the Dec1-PPARγ axis was reprogrammed in PAH. Further evidence confirmed that Dec1 was the missing link among the hypoxic environment, PPARγ dysfunctions, and endothelial phenotypic abnormalities.

Despite the underlying mechanisms are not entirely understood, OSA has been well-known as an important risk factor for PAH, which is closely relevant to the hypoxia-induced PA remodeling. The hypoxia-associated genes were screened in the public PAH database to fully explore the role of hypoxic signaling in HPH. The first hint provided by our bioinformatic studies showed a possible connection between the Dec1-PPARγ axis and PAH. In view of the dysregulation of the Dec1-PPARγ axis in PAH database (Figures 1C,D), we asked whether this hypoxic signaling was also reprogrammed in HPH. It is well established that hypoxic reprogramming induced by HIF-1α initiates intracellular signaling cascades leading to vascular remodeling, such as Notch, NF-κB, AKT, and PPARγ signaling, which play critical roles in PAH (Gong et al., 2011). As expected, we observed an increased Dec1 expression and decreased PPARγ expression in plasma of the OSA patients with PAH compared with OSA patients without PAH. This change was in a negative correlation (Figure 1I), suggesting that hypoxic Dec1 would be involved in downregulating of PPARγ. To the best of our knowledge, this is the first study demonstrating a role of the Dec1-PPARγ axis in the pulmonary vasculature of HPH.

A close relationship was found between hypoxia and the insufficient PPARγ signaling in the pathogenesis of PAH, although the mechanism is still unclear. This concept is supported by that genetic mutations or stimuli of PPARγ regulates development of PAH both *in vivo* and *in vitro* (Hansmann et al., 2008; Gong et al., 2011; Calvier et al., 2017, 2019). Several studies observed the spontaneous PAH developing in mice with conditional deletion of PPARγ in PASMCs and PAECs (Hansmann et al., 2008; Guignabert et al., 2009). Other experiments demonstrated that activation of PPARγ ligands by rosiglitazone attenuated pulmonary hypertension *via* suppressing oxidative, insulin resistance, and proliferative signals in mouse models treated with chronic hypoxia or apoE knockout mice fed a high-fat diet (Crossno et al., 2007; Hansmann et al., 2007; Nisbet et al., 2010). More recent investigations tried to provide the possible mechanism of how hypoxia downregulated the PPARγ expression (Gong et al., 2011; Yao et al., 2021). For example, Kaizheng et al. showed that hypoxia-induced downregulation of PPARγ expression is TGF-β-dependent through increasing Smad2/3/4 and HDAC1 binding at the transcriptional level (Gong et al., 2011). In the present study, we found that insufficient PPARγ signaling is dependent on the induction of Dec1 under hypoxia. These findings are very appealing as it provides an initial insight into the mechanism of which genes with oxygen availability exert a major effect in hypoxic endothelial dysfunctions.

**FIGURE 7:**
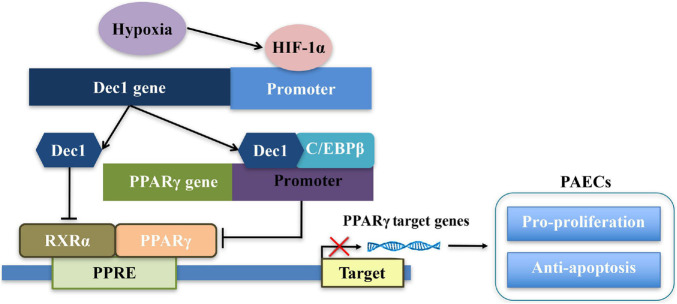
Schema of the signaling pathways leading to disruption of Dec1-PPARγ axis in PAECs in response to hypoxia.

The effects and mechanisms of Dec1 on regulation of PPARγ have been elaborated (Figure 7; Cho et al., 2009; Park and Park, 2012; Noshiro et al., 2018). First, Dec1 occupies the specific region of PPARγ promoter and negatively regulates PPARγ expression by interacting with CCAAT/Enhancer Binding Protein β (C/EBPβ) (Park and Park, 2012). Second, as a transcriptional regulator, Dec1 directly binds to the E-boxes or Sp1 sites of the target genes, therefore suppressing and promoting their transcriptions, respectively (Kato et al., 2014). Dec1 transcriptionally represses RXRα, which is a crucial heterodimer partner of PPARγ, via binding to the E-boxes of RXRα promoter (Cho et al., 2009; Noshiro et al., 2018). Third, peroxisome proliferator-activated receptor-γ coactivator-1α (PGC-1α) is also a key regulator of oxidative metabolism and mitochondrial function in PAH and hypoxia-induced repression of PGC-1α plays a critical role in PAH development (Ye et al., 2016). Dec1 could also directly target the E-box of the PGC-1α core promoter and transcriptionally represses PGC-1α (Hsiao et al., 2009; LaGory et al., 2015). Consistent with these studies, we found that PPARγ expression was not only significantly downregulated by induction of Dec1 but also obviously upregulated *via* silencing of Dec1 in PAECs (Figures 6A,B). Notably, we found that hypoxia-induced downregulation of PPARγ could be restored by inhibition of Dec1 (Figure 6A), which indicated that Dec1 was essential for the PPARγ deficiency under hypoxia.

Although Dec1 is well known as an oncogene in tumors (Nakamura et al., 2008; Feige et al., 2011; Sato et al., 2016; Jia et al., 2018), the present study also adds to a growing body of molecular evidence linking Dec1 to hypoxia-associated diseases (Nishiyama et al., 2012; Wu et al., 2017; Huang et al., 2018; Nakashima et al., 2018; Le et al., 2019). For example, Hue et al. previously reported that Dec1 expression was significantly upregulated in human cardiac hypertrophy and myocardial infarction, while Dec1 deficiency suppressed cardiac perivascular fibrosis and attenuated expressions of p21, TGF-β1, pSmad3, and TNF-α in transverse aortic constriction treated mouse models (Le et al., 2019). Although the potential mechanism remained to be specified, this study provided key findings regarding Dec1 in transverse aortic constriction induced by hypoxia, inflammation, and fibrosis in cardiovascular diseases. In addition, Ayumu et al. demonstrated that Dec1-deficient mice showed decreased blood pressure *in vivo*, and mechanistically, Dec1 suppressed activity of the Na^+^/K^+^-ATPase by transcriptional repression of Atp1b1 (Prabhakar et al., 2020). It is worth noting that this study also provided indirect evidence that Dec1 might have an important impact on regulating the contraction of pulmonary artery vascular smooth muscle and led to PAH. Owing to the critical roles of hypoxia in blood pressure, it is likely that besides PAH, hypoxic Dec1 may be involved in hypoxic hypertension. Along with these findings, we described Dec1 upregulation to be a key mediator regulating proliferation and apoptosis of PAECs *via* inhibition of PPARγ under hypoxia conditions. All these findings provided therapeutic interest that hypoxia enhanced Dec1 signaling, which in turn induced genetic reprogramming and promoted development of cardiovascular diseases.

The balance of pro-apoptotic and anti-apoptotic factors regulates cell death and survival. The anti-apoptotic effect of Dec1 has been observed under various environmental stimuli in cell lines and animal models, specifically in the field of tumor (Seino et al., 2015; Li et al., 2016; Sato et al., 2016; Jia et al., 2018). However, the role of Dec1 in endothelial apoptosis under hypoxia is poorly understood. For the first time, this work revealed the anti-apoptotic role of Dec1 in hypoxic PAECs by negatively regulating PPARγ expression. Hypoxia reduced number of apoptotic PAECs, which was restored by Dec1 knockdown (Figure 5D). Emerging evidence suggested that the Bax/Bcl-2 ratio and cleaved caspase 3 expression contribute to cell viability to apoptotic stimuli. Changes in the Bax/Bcl-2 ratio and cleaved caspase 3 expression were found in Dec1-overexpressed or silenced PAECs (Figures 5E,F), indicating the crucial role of Dec1 in the apoptotic sensitivity of endothelial cells under hypoxia. A recent study also revealed that Dec1 transcriptionally upregulates Survivin, a family member of the “anti-apoptotic factors,” and facilitates the survival of gastric cancer cells under hypoxia (Jia et al., 2018). Xin et al. described Dec1 as a prosurvival factor because it upregulates clusterin, a cytoprotective protein that guards against genotoxic stresses to reduce DNA damage-induced apoptotic response (Ming et al., 2018). Therefore, Dec1 might be one of the key anti-apoptotic factors in hypoxic reprogramming during PAH development.

In addition to apoptosis resistance, the increased proliferation of vascular cells contributes to the progressive remodeling of pulmonary arteries and the formation of PAH (Kim et al., 2010). Recent evidence indicates that PPARγ dysfunction plays a crucial role in the excessive PASMC proliferation of hypoxic and other types of PAH (Ameshima et al., 2003; Kim et al., 2010; Xu et al., 2017), and further reveals the effect of PPARγ on anti-proliferation (Hansmann et al., 2008; Calvier et al., 2017, 2019). For instance, the TGF-β1-promoted VSMC proliferation is PPARγ dependent; on the one hand, PPARγ deletion activates TGF-β1 signaling, and on the other hand, TGF-β1 suppresses PPARγ by inducing miR-130a/301b (Calvier et al., 2017). However, the upstream mediators that downregulate PPARγ are yet to be defined. Hypoxia increases Dec1 (Wykoff et al., 2000) but decreases PPARγ (Kim et al., 2010), a transcriptional target of Dec1 (Park and Park, 2012). Therefore, Dec1-induced PPARγ repression might be important in HPH pathogenesis. As shown in Figures 2, 3, PPARγ expression was significantly decreased by Dec1 overexpression in PAH-PAECs and hypoxia-treated PAECs but was enhanced by Dec1 knockdown (Figures 6A,B). These data clarified the hypothesis that hypoxic Dec1 facilitates PAH by inhibiting PPARγ.

Hypoxia is a well-known trigger of vascular remodeling, and PPARγ deficiency is a crucial contributor in PAH development. This study presents the missing link between hypoxia-responsive genes and insufficient PPARγ signaling in PAH pathogenesis. The findings further reveal a causative role of Dec1-PPARγ axis in the imbalance of endothelial proliferation and apoptosis under hypoxia. From the clinical point of view, the Dec1-PPARγ axis might be a novel therapeutic strategy for PAH, especially in HPH.

## Data Availability Statement

The original contributions presented in the study are included in the article/supplementary material, further inquiries can be directed to the corresponding author/s.

## Ethics Statement

The studies involving human participants were reviewed and approved by Ethics Committee of Shandong Provincial Hospital Affiliated to Shandong First Medical University. The patients/participants provided their written informed consent to participate in this study. Written informed consent was obtained from the individual(s) for the publication of any potentially identifiable images or data included in this article.

## Author Contributions

XL, MX, and MZ designed the study. QM, HZ, ZT, RX, XW, FZ, and YQ were responsible for the experimental work. CL and WQ participated in the bioinformatic analysis. XL and MX were contributed to writing and/or revision of the manuscript. XL, MX, CL, MZ, and FX provided funding of this research. All authors approved the submitted version.

## Conflict of Interest

The authors declare that the research was conducted in the absence of any commercial or financial relationships that could be construed as a potential conflict of interest.

## Publisher’s Note

All claims expressed in this article are solely those of the authors and do not necessarily represent those of their affiliated organizations, or those of the publisher, the editors and the reviewers. Any product that may be evaluated in this article, or claim that may be made by its manufacturer, is not guaranteed or endorsed by the publisher.

## Footnotes

1
https://string-db.org

## References

[B1] Adir Y. Humbert M. Chaouat A. (2021). Sleep-related breathing disorders and pulmonary hypertension. *Eur. Respir. J.* 57:2002258. 10.1183/13993003.02258-2020 32747397

[B2] Alastalo T. P. Li M. Perez Vde J. Pham D. Sawada H. Wang J. K. (2011). Disruption of PPARγ/β-catenin-mediated regulation of apelin impairs BMP-induced mouse and human pulmonary arterial EC survival. *J. Clin. Invest.* 121 3735–3746. 10.1172/JCI43382 21821917 PMC3163943

[B3] Ameshima S. Golpon H. Cool C. D. Chan D. Vandivier R. W. Gardai S. J. (2003). Peroxisome proliferator-activated receptor gamma (PPARgamma) expression is decreased in pulmonary hypertension and affects endothelial cell growth. *Circ. Res.* 92 1162–1169. 10.1161/01.RES.0000073585.50092.14 12714563

[B4] Bauters F. Rietzschel E. R. Hertegonne K. Chirinos J. A. (2016). The link between obstructive sleep apnea and cardiovascular disease. *Curr. Atheroscl. Rep.* 18:1. 10.1007/s11883-015-0556-z 26710793

[B5] Benjafield A. Ayas N. Eastwood P. Heinzer R. Ip M. Morrell M. (2019). Estimation of the global prevalence and burden of obstructive sleep apnoea: a literature-based analysis. *Lancet Respir. Med.* 7 687–698. 10.1016/S2213-2600(19)30198-5 31300334 PMC7007763

[B6] Calvier L. Boucher P. Herz J. Hansmann G. (2019). LRP1 deficiency in vascular SMC leads to pulmonary arterial hypertension that is reversed by PPARγ activation. *Circ. Res.* 124 1778–1785. 10.1161/CIRCRESAHA.119.315088 31023188 PMC6554044

[B7] Calvier L. Chouvarine P. Legchenko E. Hoffmann N. Geldner J. Borchert P. (2017). PPARγ links BMP2 and TGFβ1 pathways in vascular smooth muscle cells, regulating cell proliferation and glucose metabolism. *Cell Metab.* 25 1118–1134.e7. 10.1016/j.cmet.2017.03.011 28467929

[B8] Cho Y. Noshiro M. Choi M. Morita K. Kawamoto T. Fujimoto K. (2009). The basic helix-loop-helix proteins differentiated embryo chondrocyte (DEC) 1 and DEC2 function as corepressors of retinoid X receptors. *Mol. Pharmacol.* 76 1360–1369. 10.1124/mol.109.057000 19786558

[B9] Comhair S. Xu W. Mavrakis L. Aldred M. A. Asosingh K. Erzurum S. C. (2012). Human primary lung endothelial cells in culture. *Am. J. Respir. Cell Mol. Biol.* 46 723–730. 10.1165/rcmb.2011-0416TE 22427538 PMC3380284

[B10] Crossno J. T. Jr. Garat C. V. Reusch J. E. Morris K. G. Dempsey E. C. Mcmurtry I. F. (2007). Rosiglitazone attenuates hypoxia-induced pulmonary arterial remodeling. *Am. J. Physiol. Lung Cell Mol. Physiol.* 292 L885–L897. 10.1152/ajplung.00258.2006 17189321

[B11] Feige E. Yokoyama S. Levy C. Khaled M. Igras V. Lin R. (2011). Hypoxia-induced transcriptional repression of the melanoma-associated oncogene MITF. *Proc. Natl. Acad. Sci.U.S.A.* 108 E924–E933. 10.1073/pnas.1106351108 21949374 PMC3203758

[B12] Gong K. Xing D. Li P. Aksut B. Ambalavanan N. Yang Q. (2011). Hypoxia induces downregulation of PPAR-γ in isolated pulmonary arterial smooth muscle cells and in rat lung via transforming growth factor-β signaling. *Am. J. Physiol. Lung Cell Mol. Physiol.* 301 L899–L907. 10.1152/ajplung.00062.2011 21926264 PMC3233825

[B13] Guignabert C. Alvira C. M. Alastalo T. P. Sawada H. Hansmann G. Zhao M. (2009). Tie2-mediated loss of peroxisome proliferator-activated receptor-gamma in mice causes PDGF receptor-beta-dependent pulmonary arterial muscularization. *Am. J. Physiol. Lung Cell Mol. Physiol.* 297 L1082–L1090. 10.1152/ajplung.00199.2009 19801450 PMC2793182

[B14] Hansmann G. de Jesus Perez V. A. Alastalo T. P. Alvira C. M. Guignabert C. Bekker J. M. (2008). An antiproliferative BMP-2/PPARγ/apoE axis in human and murine SMCs and its role in pulmonary hypertension. *J. Clin. Invest.* 118 1846–1857. 10.1172/JCI32503 18382765 PMC2276393

[B15] Hansmann G. Wagner R. A. Schellong S. Perez V. A. Urashima T. Wang L. (2007). Pulmonary arterial hypertension is linked to insulin resistance and reversed by peroxisome proliferator-activated receptor-gamma activation. *Circulation* 115 1275–1284. 10.1161/CIRCULATIONAHA.106.663120 17339547

[B16] Houten B. V. (2015). Pulmonary arterial hypertension is associated with oxidative stress–induced genome instability. *Am. J. Respir. Crit. Care Med.* 192 129–130. 10.1164/rccm.201505-0904ED 26177169 PMC4532829

[B17] Hsiao S. P. Huang K. M. Chang H. Y. Chen S. L. (2009). P/CAF rescues the Bhlhe40-mediated repression of MyoD transactivation. *Biochem. J.* 422 343–352. 10.1042/BJ20090072 19522704

[B18] Huang Y. Lai X. Hu L. Lei C. Lan X. Zhang C. (2018). Over-expression of DEC1 inhibits myogenic differentiation by modulating MyoG activity in bovine satellite cell. *J. Cell. Physiol.* 233 9365–9374. 10.1002/jcp.26471 29350420

[B19] Humbert M. Guignabert C. Bonnet S. Dorfmüller P. Klinger J. R. Nicolls M. R. (2018). Pathology and pathobiology of pulmonary hypertension: state of the art and research perspectives. *Eur. Respir. J.* 53:1801887. 10.1183/13993003.01887-2018 30545970 PMC6351340

[B20] Jia Y. Hu R. Li P. Zheng Y. Wang Y. Ma X. (2018). DEC1 is required for anti-apoptotic activity of gastric cancer cells under hypoxia by promoting Survivin expression. *Gastr. Cancer* 21 632–642. 10.1007/s10120-017-0780-z 29204860

[B21] Kato Y. Kawamoto T. Fujimoto K. Noshiro M. (2014). DEC1/STRA13/SHARP2 and DEC2/SHARP1 coordinate physiological processes, including circadian rhythms in response to environmental stimuli. *Curr. Top. Dev. Biol.* 110 339–372. 10.1016/B978-0-12-405943-6.00010-5 25248482

[B22] Kim E. K. Lee J. H. Oh Y. M. Lee Y. S. Lee S. D. (2010). Rosiglitazone attenuates hypoxia-induced pulmonary arterial hypertension in rats. *Respirology* 15 659–668. 10.1111/j.1440-1843.2010.01756.x 20546541

[B23] LaGory E. L. Wu C. Taniguchi C. M. Ding C. C. Chi J. T. Von Eyben R. (2015). Suppression of PGC-1alpha is critical for reprogramming oxidative metabolism in renal cell carcinoma. *Cell Rep.* 12 116–127. 10.1016/j.celrep.2015.06.006 26119730 PMC4518559

[B24] Le H. T. Sato F. Kohsaka A. Bhawal U. K. Nakao T. Muragaki Y. (2019). Dec1 deficiency suppresses cardiac perivascular fibrosis induced by transverse aortic constriction. *Int. J. Mol. Sci.* 20:4967. 10.3390/ijms20194967 31597354 PMC6802004

[B25] Li X. Xu F. Meng Q. Gong N. Xia M. (2020). Long noncoding RNA DLEU2 predicts a poor prognosis and enhances malignant properties in laryngeal squamous cell carcinoma through the miR-30c-5p/PIK3CD/Akt axis. *Cell Death Dis.* 11:472. 10.1038/s41419-020-2581-2 32555190 PMC7303144

[B26] Li X. M. Lin W. Wang J. Zhang W. Yin A. A. Huang Y. (2016). Dec1 expression predicts prognosis and the response to temozolomide chemotherapy in patients with glioma. *Mol. Med. Rep.* 14 5626–5636. 10.3892/mmr.2016.5921 27840944

[B27] Ming X. Bao C. Hong T. Yang Y. Chen X. Jung Y. S. (2018). Clusterin, a novel DEC1 target, modulates dna damage-mediated cell death. *Mol. Cancer Res.* 16 1641–1651. 10.1158/1541-7786.MCR-18-0070 30002194

[B28] Montaigne D. Butruille L. Staels B. (2021). PPAR control of metabolism and cardiovascular functions. *Nat. Rev. Cardiol.* 10.1038/s41569-021-00569-6 34127848

[B29] Nakamura H. Tanimoto K. Hiyama K. Yunokawa M. Kawamoto T. Kato Y. (2008). Human mismatch repair gene, MLH1, is transcriptionally repressed by the hypoxia-inducible transcription factors, DEC1 and DEC2. *Oncogene* 27 4200–4209. 10.1038/onc.2008.58 18345027

[B30] Nakashima A. Kawamoto T. Noshiro M. Ueno T. Doi S. Honda K. (2018). Dec1 and CLOCK Regulate Na(+)/K(+)-ATPase β1 subunit expression and blood pressure. *Hypertension* 72 746–754. 10.1161/HYPERTENSIONAHA.118.11075 30012868

[B31] Nisbet R. E. Bland J. M. Kleinhenz D. J. Mitchell P. O. Walp E. R. Sutliff R. L. (2010). Rosiglitazone attenuates chronic hypoxia-induced pulmonary hypertension in a mouse model. *Am. J. Respir. Cell Mol. Biol.* 42 482–490. 10.1165/rcmb.2008-0132OC 19520921 PMC2848739

[B32] Nishiyama Y. Goda N. Kanai M. Niwa D. Osanai K. Yamamoto Y. (2012). HIF-1α induction suppresses excessive lipid accumulation in alcoholic fatty liver in mice. *J. Hepatol.* 56 441–447. 10.1016/j.jhep.2011.07.024 21896344

[B33] Noshiro M. Kawamoto T. Nakashima A. Ozaki N. Ueno T. Saeki M. (2018). Deficiency of the basic helix-loop-helix transcription factor DEC1 prevents obesity induced by a high-fat diet in mice. *Genes Cells* 10.1111/gtc.12607 29968353

[B34] Park Y. K. Park H. (2012). Differentiated embryo chondrocyte 1 (DEC1) represses PPARgamma2 gene through interacting with CCAAT/enhancer binding protein beta (C/EBPbeta). *Mol. Cells* 33 575–581. 10.1007/s10059-012-0002-9 22610404 PMC3887761

[B35] Polikandriotis J. A. Mazzella L. J. Rupnow H. L. Hart C. M. (2005). Peroxisome proliferator-activated receptor gamma ligands stimulate endothelial nitric oxide production through distinct peroxisome proliferator-activated receptor gamma-dependent mechanisms. *Arteriosc. Thromb. Vasc. Biol.* 25 1810–1816. 10.1161/01.ATV.0000177805.65864.d4 16020752

[B36] Prabhakar N. Peng Y. Nanduri J. (2020). Hypoxia-inducible factors and obstructive sleep apnea. *J. Clin. Invest.* 130 5042–5051. 10.1172/JCI137560 32730232 PMC7524484

[B37] Rawal H. Angadi U. Mondal T. (2021). TEnGExA: an R package based tool for tissue enrichment and gene expression analysis. *Brief. Bioinform.* 22:bbaa221. 10.1093/bib/bbaa221 32960209

[B38] Reyes-Palomares A. Gu M. Grubert F. Berest I. Zaugg J. B. (2020). Remodeling of active endothelial enhancers is associated with aberrant gene-regulatory networks in pulmonary arterial hypertension. *Nat. Commun.* 11:1673. 10.1038/s41467-020-15463-x 32245974 PMC7125148

[B39] Sato F. Bhawal U. Yoshimura T. Muragaki Y. (2016). DEC1 and DEC2 crosstalk between circadian rhythm and tumor progression. *J. Cancer* 7 153–159. 10.7150/jca.13748 26819638 PMC4716847

[B40] Sehgal P. Mukhopadhyay S. (2007). Dysfunctional intracellular trafficking in the pathobiology of pulmonary arterial hypertension. *Am. J. Respir. Cell Mol. Biol.* 37 31–37. 10.1165/rcmb.2007-0066TR 17363775 PMC1899345

[B41] Seino H. Wu Y. Morohashi S. Kawamoto T. Fujimoto K. Kato Y. (2015). Basic helix-loop-helix transcription factor DEC1 regulates the cisplatin-induced apoptotic pathway of human esophageal cancer cells. *Biomed. Res.* 36 89–96. 10.2220/biomedres.36.89 25876658

[B42] Simonneau G. Gatzoulis M. A. Adatia I. Celermajer D. Denton C. Ghofrani A. (2014). Updated clinical classification of pulmonary hypertension. *J. Am. Coll. Cardiol.* 62(25 Suppl) D34–D41. 10.1016/j.jacc.2013.10.029 24355639

[B43] Steiner M. K. Syrkina O. L. Kolliputi N. Mark E. J. Hales C. A. Waxman A. B. (2009). Interleukin-6 overexpression induces pulmonary hypertension. *Circ. Res.* 104 236–244,228following244. 10.1161/CIRCRESAHA.108.182014 19074475 PMC5482545

[B44] Stenmark K. R. Fagan K. A. Frid M. G. (2006). Hypoxia-induced pulmonary vascular remodeling: cellular and molecular mechanisms. *Circ. Res.* 99 675–691. 10.1161/01.RES.0000243584.45145.3f 17008597

[B45] Szklarczyk D. Gable A. L. Lyon D. Junge A. Wyder S. Huerta-Cepas J. (2019). STRING v11: protein-protein association networks with increased coverage, supporting functional discovery in genome-wide experimental datasets. *Nucleic Acids Res.* 47 D607–D613. 10.1093/nar/gky1131 30476243 PMC6323986

[B46] Teng X. Zhang Z. He G. Yang L. Li F. (2012). Validation of reference genes for quantitative expression analysis by real-time RT-PCR in four lepidopteran insects. *J. Insect Sci.* 12 1–17. 10.1673/031.012.6001 22938136 PMC3481461

[B47] Wang Y. Pandey R. N. York A. J. Mallela J. Nichols W. C. Hu Y. C. (2019). The EYA3 tyrosine phosphatase activity promotes pulmonary vascular remodeling in pulmonary arterial hypertension. *Nat. Commun.* 10:4143. 10.1038/s41467-019-12226-1 31515519 PMC6742632

[B48] Wickham H. (2009). *Ggplot2: Elegant Graphics for Data Analysis.* Cham: Springer. 10.1007/978-0-387-98141-3

[B49] Wu L. H. Cheng W. Yu M. He B. M. Sun H. Chen Q. (2017). Nr3C1-Bhlhb2 axis dysregulation is involved in the development of attention deficit hyperactivity. *Mol. Neurobiol.* 54 1196–1212. 10.1007/s12035-015-9679-z 26820676 PMC5310568

[B50] Wykoff C. C. Pugh C. W. Maxwell P. H. Harris A. L. Ratcliffe P. J. (2000). Identification of novel hypoxia dependent and independent target genes of the von Hippel-Lindau (VHL) tumour suppressor by mRNA differential expression profiling. *Oncogene* 19 6297–6305. 10.1038/sj.onc.1204012 11175344

[B51] Xu Y. Gu Q. Liu N. Yan Y. Yang X. Hao Y. (2017). PPARgamma alleviates right ventricular failure secondary to pulmonary arterial hypertension in rats. *Int. Heart J.* 58 948–956. 10.1536/ihj.16-591 29151490

[B52] Yao D. He Q. Sun J. Cai L. Wei J. Cai G. (2021). FGF21 attenuates hypoxia-induced dysfunction and inflammation in HPAECs via the microRNA-27b-mediated PPARγ pathway. *Int. J. Mol. Med.* 47:116. 10.3892/ijmm.2021.4949 33907846 PMC8083827

[B53] Ye J. X. Wang S. S. Ge M. Wang D. J. (2016). Suppression of endothelial PGC-1alpha is associated with hypoxia-induced endothelial dysfunction and provides a new therapeutic target in pulmonary arterial hypertension. *Am. J. Physiol. Lung Cell Mol. Physiol.* 310 L1233–L1242. 10.1152/ajplung.00356.2015 27084848

[B54] Yu G. Wang L. G. Han Y. He Q. Y. (2012). clusterProfiler: an R package for comparing biological themes among gene clusters. *Omics* 16 284–287. 10.1089/omi.2011.0118 22455463 PMC3339379

[B55] Zhao M. Sun D. Li X. Xu Y. Zhang H. Qin Y. (2017). Overexpression of long noncoding RNA PEG10 promotes proliferation, invasion and metastasis of hypopharyngeal squamous cell carcinoma. *Oncol. Lett.* 14 2919–2925. 10.3892/ol.2017.6498 28928830 PMC5588139

